# Molecular and Morphological Evidence Challenges the Records of the Extant Liverwort *Ptilidium pulcherrimum* in Eocene Baltic Amber

**DOI:** 10.1371/journal.pone.0140977

**Published:** 2015-11-04

**Authors:** Jochen Heinrichs, Armin Scheben, Gaik Ee Lee, Jiří Váňa, Alfons Schäfer-Verwimp, Michael Krings, Alexander R. Schmidt

**Affiliations:** 1 Department of Biology and Geobio-Center, University of Munich (LMU), Munich, Germany; 2 Department of Botany, Charles University, Praha, Czech Republic; 3 Mittlere Letten 11, Herdwangen-Schönach, Germany; 4 Department of Earth and Environmental Sciences, University of Munich (LMU), and Bavarian State Collection for Palaeontology and Geology, Munich, Germany; 5 Department of Geobiology, University of Göttingen, Göttingen, Germany; Field Museum of Natural History, UNITED STATES

## Abstract

Preservation of liverworts in amber, a fossilized tree resin, is often exquisite. Twenty-three fossil species of liverworts have been described to date from Eocene (35–50 Ma) Baltic amber. In addition, two inclusions have been assigned to the extant species *Ptilidium pulcherrimum* (Ptilidiales or Porellales). However, the presence of the boreal *P*. *pulcherrimum* in the subtropical or warm-temperate Baltic amber forest challenges the phytogeographical interpretation of the Eocene flora. A re-investigation of one of the fossils believed to be *P*. *pulcherrimum* reveals that this specimen in fact represents the first fossil evidence of the genus *Tetralophozia*, and thus is re-described here as *Tetralophozia groehnii* sp. nov. A second fossil initially assigned to *P*. *pulcherrimum* is apparently lost, and can be reassessed only based on the original description and illustrations. This fossil is morphologically similar to the extant North Pacific endemic *Ptilidium californicum*, rather than *P*. *pulcherrimum*. Divergence time estimates based on chloroplast DNA sequences provide evidence of a Miocene origin of *P*. *pulcherrimum*, and thus also argue against the presence of this taxon in the Eocene. *Ptilidium californicum* originated 25–43 Ma ago. As a result, we cannot rule out that the Eocene fossil belongs to *P*. *californicum*. Alternatively, the fossil might represent a stem lineage element of *Ptilidium* or an early crown group species with morphological similarities to *P*. *californicum*.

## Introduction

Liverworts belong to the oldest lineages of plants on land [1–2]; however, their exact position in the tree of life remains unclear [3–8]. The reconstruction of early land plant evolution is generally hampered by the incompleteness of the fossil record [9–10]. Moreover, widely differing hypotheses have been offered on the age of the land plant crown group [11] and their closest relatives [12]. In spite of these limitations, considerable progress has been made in recent years regarding the reconstruction of the evolutionary history of the liverworts. DNA-based divergence time estimates suggest a Paleozoic origin of several main liverwort lineages [13–14]. Moreover, the crown group diversification of most extant genera appears to have started during the Cretaceous or Cenozoic, concomitant with the diversification of angiosperms [15–18]. While the general pattern of liverwort diversification is well-supported by the available dating studies, age estimates fluctuate considerably, due in part to deviating node calibrations based on fossil evidence. This makes it all the more important to study the fossil record and determine the usability of fossils in constraining molecular dating analyses [19].

The Paleozoic and early Mesozoic fossil record of liverworts is meagre and most specimens are ill-preserved [20–21]. The situation changes for the better in the late Mesozoic [22–23]. Finally, Cenozoic strata have yielded numerous liverwort fossils in faithful cellular and ultrastructural preservation, most in the form of amber inclusions [24]. Amber, a solidified gymnosperm or angiosperm tree resin, is an excellent preservation medium that provides detailed insights into the diversity of soft-bodied organisms such as liverworts [25]. Approximately 200 amber deposits are known worldwide, the most widely known of which are located in the Baltic region [26]. Baltic amber has been dated as Eocene [27–28]. It was already mentioned by Pliny the Elder (Naturalis Historia, book 37), who considered it a type of spruce resin [29]. This “classic” hypothesis is largely accurate; however, the amber-producing tree was likely not a spruce, but rather a representative of the Sciadopityaceae [30] growing in a subtropical or warm-temperate mixed forest [31] together with several representatives of Cupressaceae and Pinaceae, as well as angiosperms, especially Fagaceae [28,32].

Baltic amber inclusions have been studied since the 18^th^ century [33]; the first liverwort fossils in Baltic amber have been described in 1845 [34]. Several authors have since documented additional taxa or revised earlier classifications [35–38]. A comprehensive monograph [39] formally accepts 22 species of liverworts from Baltic amber, including *Ptilidium pulcherrimum* (Weber) Vain. *Ptilidium pulcherrimum* belongs to a small genus of terrestrial or epiphytic liverworts characterized by exclusively lateral branching and deeply lobed, incubous leaves with long uniseriate marginal cilia [40]. Three extant species are distinguished based on morphology: the North Pacific endemic *Ptilidium californicum* (Austin) Pearson; and the two widespread circumboreal *P*. *ciliare* (L.) Hampe and *P*. *pulcherrimum*. Leaves of *P*. *californicum* are sparsely ciliate or entire-margined, while the leaf lobes in *P*. *ciliare* and *P*. *pulcherrimum* are densely ciliate. Molecular phylogenies place *P*. *californicum* as sister to a clade containing the other two species [41]. Occurrence of the cold-temperate *P*. *pulcherrimum* in the warm Eocene [42] challenges the phytogeographical interpretation of the Baltic amber flora. Heinrichs et al. [13] questioned the conspecificity of the Eocene fossil and extant *P*. *pulcherrimum*; however, Frahm & Gröhn [43] identified a second inclusion as *P*. *pulcherrimum*, putting forth an adaptation to different climates during the evolutionary history of this species.

Here we present a reassessment of the fossils assigned to *Ptilidium pulcherrimum* based on morphological evidence and DNA sequence variation of extant *Ptilidium* specimens. We dismiss the fossils as evidence of the occurrence of *P*. *pulcherrimum* in the Eocene, and present an alternative taxonomic treatment for these amber inclusions.

## Material and Methods

### Amber fossils assigned to *Ptilidium*

The Eocene sediments that yield the majority of Baltic amber are 35–47 million years old, but some specimens are also found in strata up to 50 million-year-old [27–28]. The first piece of Baltic amber containing a liverwort inclusion assigned to *Ptilidium pulcherrimum* was published as specimen BB2379 of the Baltic amber collection of the Museum of Natural History Stuttgart (SMNS “coll. Velten”) [39]. We tried to find the specimen in the SMNS amber holdings, but were unsuccessful. Amber trader J. Velten informed us that he sold the specimen, but has no documents of who purchased it. As a result, the specimen is considered lost. We therefore base our revision of this liverwort inclusion on the description, images, and drawings in [39].

The second Baltic amber specimen assigned to *P*. *pulcherrimum* originally comes from the private amber collection of Carsten Gröhn [43], but has recently been donated to the collection of the Geological Palaeontological Institute Hamburg, Germany (GPIH 4575, Coll. Gröhn 5827, Syninclusion: *Cylindrocolea dimorpha* (Casp.) Grolle). The surface of the amber piece was polished manually with a series of wet silicon carbide abrasive papers (grit from FEPA P 600–4000, 25.8 μm to 5 μm particle size, firm Struers) to minimize light scattering during analysis. The specimen was then placed on a glass microscope slide with a drop of water applied to the upper surface of the amber, and covered with a coverslip. Inclusions were studied using a Leica M50 dissection microscope and a Carl Zeiss AxioScope A1 compound microscope, the latter equipped with a Canon 60D digital camera. Incident and transmitted light were used simultaneously. The illustrations accompanying our study represent digitally stacked photomicrographic composites obtained with the software package HeliconFocus 6.0. Drawings of the fossil were produced using a Leica DM1000 microscope equipped with a drawing tube. The liverwort was embedded in the resin in wet condition, and appears to have turned by a few degrees during fossilization. Resulting streaks in the amber exacerbate visual inspection and photography. Moreover, the inclusion has shrunken during fossilization and, as a result, is preserved in a cavity representing the original size of the plant. Refraction of light at the amber surface of the cavity obstructs the recognizability of the cell walls and surface structures, and further reduces digital image quality. However, the outlines and certain cellular details of the stem, leaf, and rhizoids in hydrated condition have been preserved as imprints on the surface of this cavity. Cell size measurements were taken from these imprints, as well as from plant fragments inside the cavity. Measurements of plant size and rhizoid diameter reflect the size of the cavity, rather than that of the shrunken plant.

The taxonomic treatment of the fossil is based on literature data on fossil and extant liverworts, as well as comparisons with herbarium specimens of liverworts housed at the Bavarian State Collection of Botany (M). Herbarium material of all three extant *Ptilidium* species was moistened and leaves were separated. Leaves were transferred into a drop of water on a microscope slide and covered with a coverslip. Images were captured digitally as described above for the fossil.

No permits were required for the described study, which complied with all relevant regulations.

### Nomenclature

The electronic version of this article in a Portable Document Format (PDF) in a work with an ISSN or ISBN will represent a published work according to the International Code of Nomenclature for algae, fungi, and plants, and hence the new names contained in the electronic publication of a PLOS ONE article are effectively published under that Code from the electronic edition alone, so there is no longer any need to provide printed copies. The online version of this work is archived and available from the following digital repositories: PubMed Central, LOCKSS.

### Divergence time estimates

The evolutionary history of *Ptilidium* was reconstructed using a subset of the chloroplast DNA marker set of [41]. One accession per *Ptilidium*-haplotype was chosen and related sequences were downloaded from GenBank (Table 1). Only those haplotypes of which complete sequence stretches are available (*trn*L intron, *trn*L 3'-exon, *trn*L-*trn*F intergenic spacer; *atp*B-*rbc*L; *trn*G-intron) were considered. *Neotrichocolea bissetii* (Mitt.) S. Hatt. was chosen as outgroup based on phylogenetic hypotheses of [44]. Sequences were aligned manually using BioEdit version 5.0.9 [45] and ambiguously aligned positions were excluded. Divergence time estimates were conducted using the BEAST package v.1.8.2 [46] and the HKY substitution model with four rate categories. The tree prior was a pure-birth (Yule) tree with Markov chain Monte Carlo (MCMC) run for 40 million generations, sampling every 10,000 generations. The first 10% of trees were discarded as burn-in, and the remaining trees were combined using TreeAnnotator [47]. Following analyses of the extent of rate heterogenity in Tracer 1.6 [48], we applied a strict clock model, calibrated with a plastid substitution rate of 5.0 x 10^−4^ substitutions/site/my from [49]. The ingroup was constrained to be monophyletic.

**Table 1:** Taxa used in divergence time estimates, including information on provenance of specimens, vouchers, and herbarium where the voucher is deposited, as well as GenBank accession numbers. Herbarium abbreviations: Göttingen University herbarium, Germany (GOET); East China Normal University herbarium (HSNU); National Museum of Nature and Science herbarium, Japan (TNS); University of British Columbia herbarium, Canada (UBC).

Taxon and locality	Voucher and herbarium	*trn*L-*trn*F	*atp*B-*rbc*L	*trn*G
***Neotrichocolea bissetii* (Mitt.) S. Hatt.**				
Japan	Inoue, Br. Sel. Exs. 563 (GOET)	HQ329973	HQ330109	HQ330244
***Ptilidium californicum* (Austin) Pearson**				
U.S.A	Schofield 114357 (UBC)	HQ329991	HQ330126	HQ330261
***Ptilidium ciliare* (L.) Hampe**				
China	Zhu 20080802–12 (HSNU)	HQ330016	HQ330151	HQ330286
Germany	Heinrichs & Schmidt 3741 (GOET)	HQ330023	HQ330158	HQ330292
Greenland	Long 13239 (JE)	HQ330026	HQ330161	HQ330295
Poland	Jedrezejko & Stebel, Hep. Pol. Exs. 204 (GOET)	HQ330030	HQ330165	HQ330299
U.S.A.	Schofield et al. 101305 (UBC)	HQ330036	HQ330171	HQ330305
U.S.A	Schofield 118109 (UBC)	HQ330051	HQ330186	HQ330320
***Ptilidium pulcherrimum* (Weber) Vain.**				
Canada	Schofield 124480 (UBC)	HQ330068	HQ330203	HQ330337
Japan	Higuchi 1197 (TNS)	HQ330077	HQ330212	HQ330345
Poland	Klama & Zarnowiec 206 (GOET)	HQ330080	HQ330215	HQ330348
U.S.A.	Schofield 116250 (UBC)	HQ330096	HQ330231	HQ330364

## Results

### Grolle & Meister fossil

**Fig 1:**
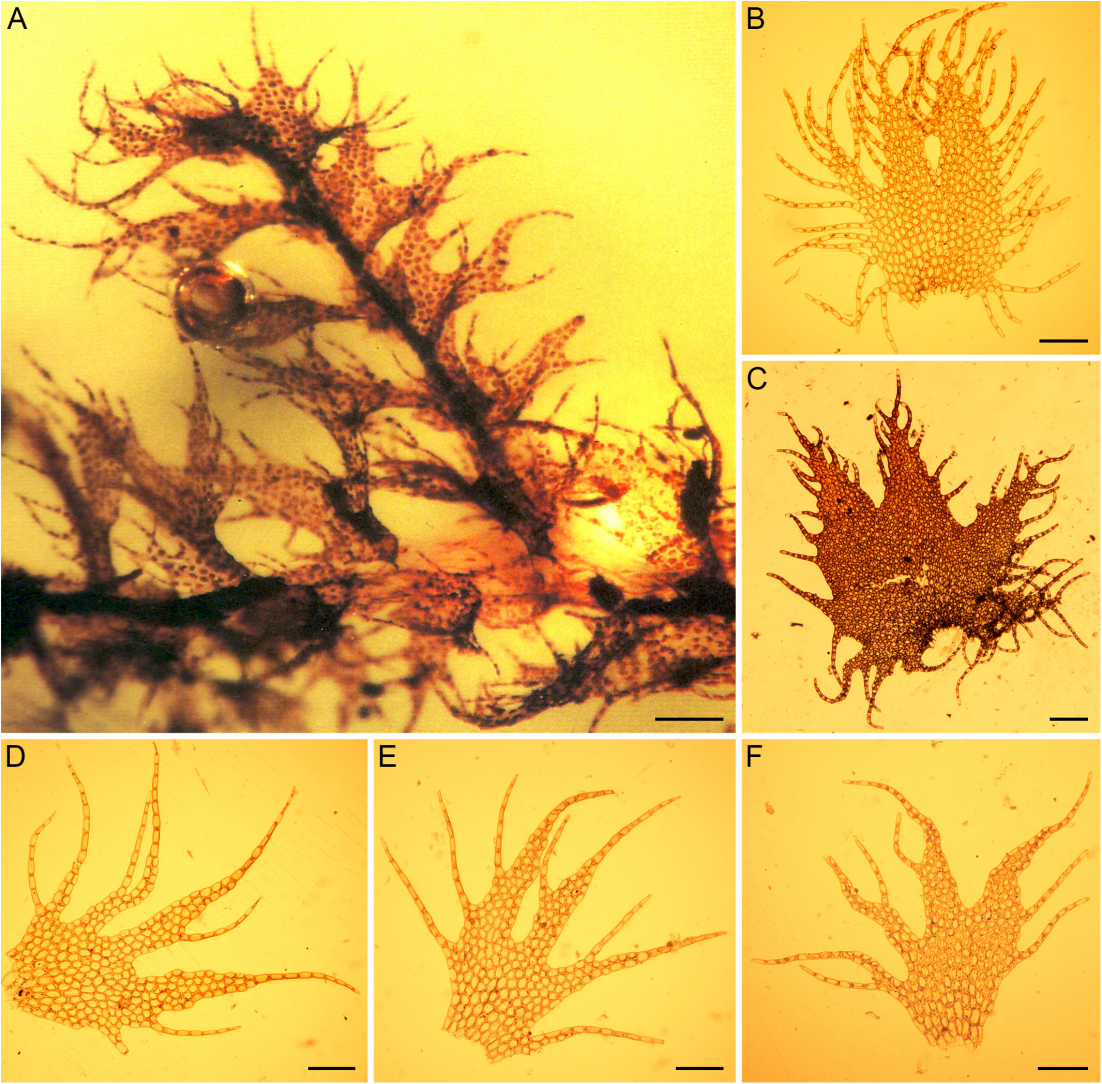
The Eocene liverwort fossil *Ptilidium* sp. (A) and leaves of extant representatives of *Ptilidium* (B–F) (scale bars 200 μm). (A) Baltic amber fossil. (B) *Ptilidium pulcherrimum*. (C) *Ptilidium ciliare*. (D–F) *Ptilidium californicum*.

According to the three published images and accompanying description, the fossil consists of a laterally branched gametophyte with deeply incised, (2–)3-lobed leaves and lobes with a few loosely arranged, long, uniseriate cilia (Fig 1A). Fasciculate rhizoids are present at the base of ciliate underleaves. Available evidence does not argue against assignment of the fossil to *Ptilidium*. However, the fossil differs from the extant *Ptilidium pulcherrimum* (Fig 1B) in the low number of leaf cilia and their loose spacing.

### Frahm & Gröhn fossil

**Fig 2:**
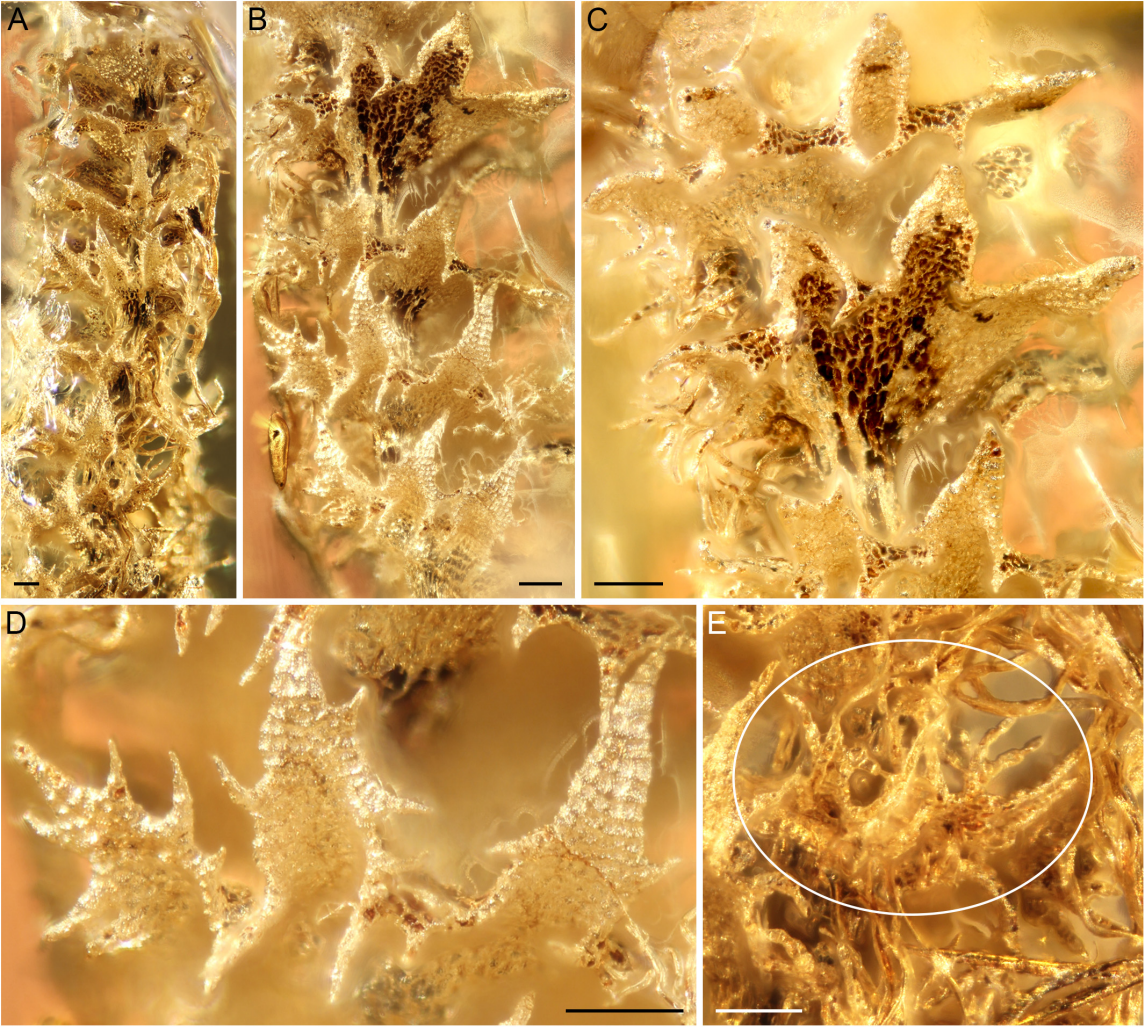
Holotype of *Tetralophozia groehnii* sp. nov. (GPIH 4575) from Baltic amber. The fossil was initially assigned to *Ptilidium* [43] but morphology is inconsistent with this genus (scale bars 100 μm). (A) Upper portion of gametophyte in lateral view. (B, C) Deeply quadrified leaves, teeth at lobe base pointing towards the plant base. (D) Impressions of leaf lobes showing leaf cells with papillose cuticle in ventral view. (E) Densely ciliate, bifid underleaf (circle).

**Fig 3:**
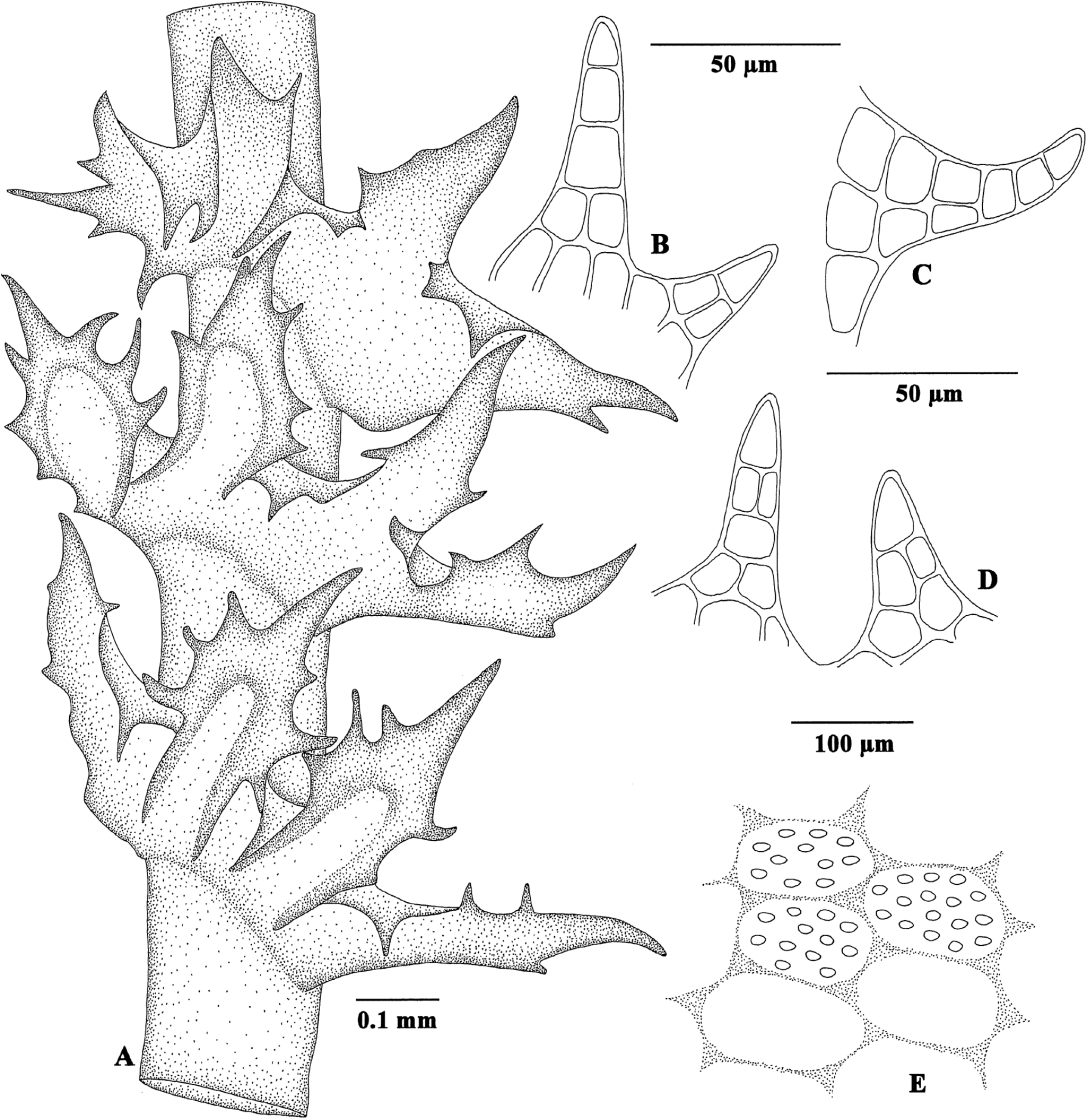
Reconstruction of *Tetralophozia groehnii* based on the holotype. (A) Portion of shoot with 3 quadrifid, succubous-subtransversely arranged leaves and partly canaliculate lobes. (B–D) Triangular to elongate-triangular, partly biseriate teeth extending from lobe margins. (E) Leaf cell pattern; papillae indicated in upper cells.

Amber piece GPIH 4575 (Gröhn 5827) includes gametophyte fragments of two different leafy liverworts, along with stellate hairs of Fagales. The fragment identified as *Ptilidium* has quadrifid, succubously-transverse leaves with canaliculate lobes and marginal teeth up to 4 cells wide and cilia (Figs 2 and 3), and thus cannot be assigned to *Ptilidium*. The specimen is re-described below as an extinct species of the extant genus *Tetralophozia* (R.M. Schust.) Schljakov:

*Tetralophozia groehnii* Heinrichs, Váňa and Schäf.-Verw., sp. nov.

Holotype: Plant fragment with quadrifid leaves in Baltic amber piece GPIH 4575 (= Coll. Gröhn 5827) of the Geological Palaeontological Institute Hamburg, Germany (Fig 2).

Diagnosis: Succubously-transverse foliated gametophyte with bifid, ciliately toothed underleaves and 2 rows of deeply quadrified leaves with canaliculate, dentate-ciliate lobes ending in uniseriate tip 1–3(–5) cells long.

Description: Sterile shoot fragment, 4.8 mm long and up to 1.2 mm wide, brown to reddish brown. Stem prostrate, round, ca. 170–250 μm in diameter, cortical cells rectangular, (25–)40–60(–90) x 12–20(–30) μm, ventrally, especially in lower sectors, with scattered colourless to pale-brownish rhizoids 12–15 μm thick; near base with a single, densely foliated young branch emerging from between rhizoids, and thus likely of ventral or ventro-lateral origin. Leaves vertically oriented, succubously-subtransverse, hand-like, deeply quadrifid, with spreading bases but upwardly bent to suberect, finger-like, abaxially canaliculate lobes, lobe margins and disc with triangular to elongate-triangular teeth 1–3(–4) cells wide and 2–8 cells long and cilia, those at lobe base often pointing towards the plant base. Teeth oriented perpendicularly to leaf margin, pointed forward, some pointing backwards, (1–)2–7 per lobe margin, straight or curved. Leaf cells ca. 8–15 x 12–25 μm, near leaf base mostly irregularly rectangular, towards apex subisodiametric or a mixture of subisodiametric and rectangular ones, walls thickened, trigones present. Cuticle with relatively large, low, globose to elliptic papillae. Underleaves conspicuous, bifid, densely ciliate, to more than 250 μm long (including cilia). Asexual reproduction not observed.

### Divergence time estimates

**Fig 4:**
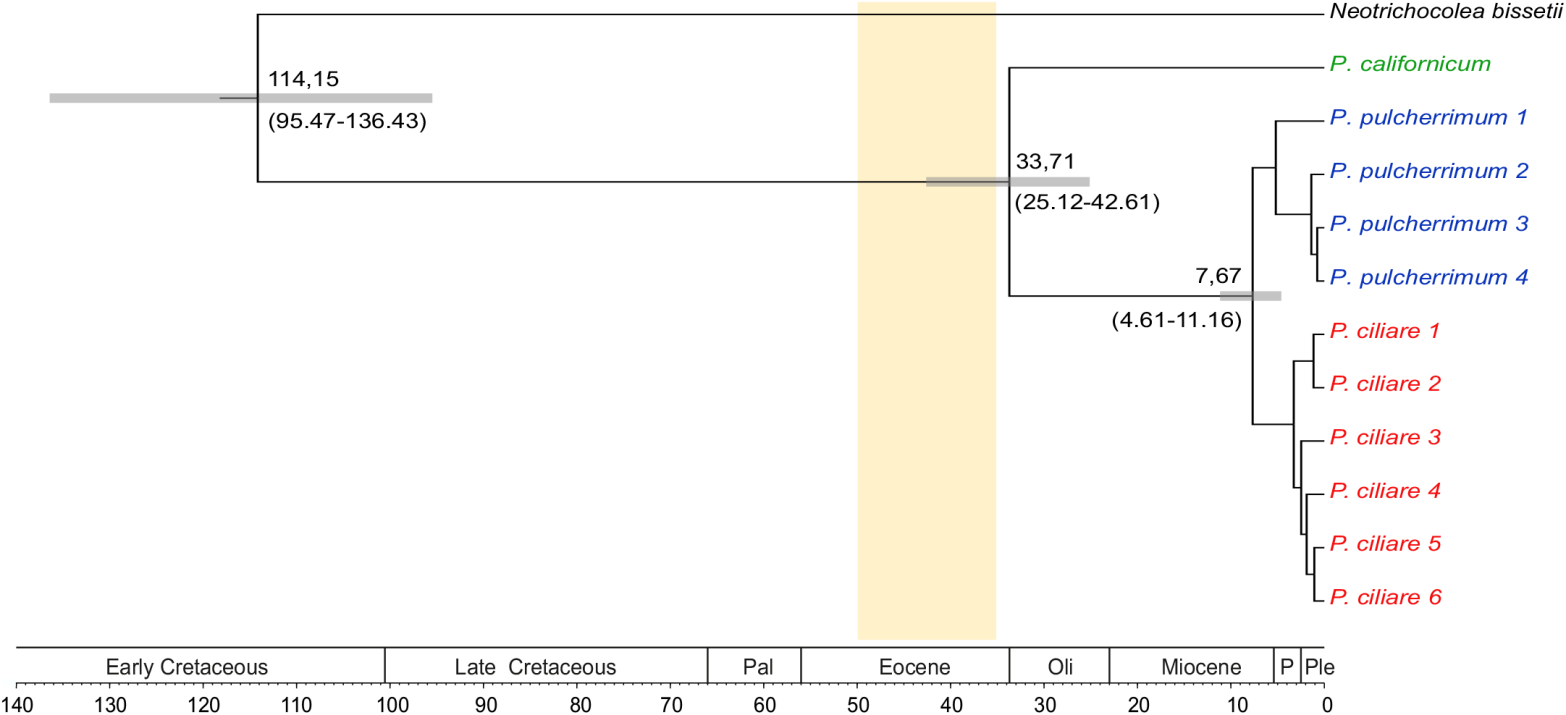
Phylogenetic chronogram for *Ptilidium* plotted against the geological time scale (Pal = Paleocene, Oli = Oligocene, P = Pliocene; Ple = Pleistocene/Holocene). Time scale shown in million years to present. Confidence age estimate intervals (split *Ptilidium-Neotrichocolea*, *Ptilidium* crown group, split *Ptilidium ciliare*/*P*. *pulcherrimum*) shown as grey horizontal bars. Vertical bar indicates time interval for Baltic amber [27–28].

*Ptilidium* separated from *Neotrichocolea* S. Hatt. during the Cretaceous (Fig 4). Crown group diversification of *Ptilidium* started in the Eocene or Oligocene (25–43 Ma). *Ptilidium californicum* is placed sister to a clade with accessions of *P*. *pulcherrimum* and *P*. *ciliare*. The separation of *P*. *pulcherrimum* and *P*. *ciliare* occurred in the Pliocene or late Miocene (4.6–11.2 Ma).

## Discussion

### Fossil record of *Ptilidium*

Only one of the two Eocene amber fossils initially assigned to *Ptilidium pulcherrimum* can actually be assigned to *Ptilidium* with some degree of confidence, whereas the second represents a different genus. While the Grolle & Meister fossil in fact appears to belong to *Ptilidium* (Fig 1A), affinities to *P*. *pulcherrimum* (Fig 1B) are dismissed. *Ptilidium pulcherrimum* is characterized by densely ciliate leaves [40,50], but the leaves of the fossil possess only a few loosely arranged cilia [39]. This arrangement of cilia resembles the North Pacific endemic *P*. *californicum* (Fig 1D–1F), which is characterized by sparsely ciliate to entire-margined leaves [41,51–52]. The fossil seems to have a slightly wider leaf sinus than extant *P*. *californicum*; however, the variation in leaf shape is difficult to determine from the images. *Ptilidium californicum* is an epiphyte that grows on the bark of gymnosperms, especially at the base of large trees or fallen logs. The species occurs southwards to northern California where it is restricted to middle-elevation forests, in a more mediterranean climate than the other two extant *Ptilidium* species [52–53]. The ecological amplitude of the present-day *P*. *californicum* does not contradict the presence of this taxon in the Baltic amber forest; however, assignment of incompletely preserved Paleogene fossils to extant species is problematic [24], especially in light of the extensive morphological homoplasy among extant taxa [54]. On the other hand, the morphological data gathered from the fossil do not suffice to reject affiliation of the fossil with *P*. *californicum*.

DNA sequence variation of extant species provides information on evolutionary history, and thus can be used to review the morphology-based classification of fossils [13,55–56]. So far, only *P*. *ciliare* and *P*. *pulcherrimum* have been included in divergence time estimates of liverworts [3,15,18]. These large-scale studies all indicate a Miocene origin for the two species, and thus cast doubt on the assignment of Eocene fossils to *P*. *pulcherrimum*. Our divergence time estimates (Fig 4) also indicate a Miocene age of *P*. *pulcherrimum*, and thus provide a further argument against the identification of the fossil by Grolle & Meister [39]. According to our reconstruction, *Ptilidium* separated from *Neotrichocolea* some 114 Ma ago, in the Early Cretaceous. This estimate corresponds well with the estimates provided by [15] (118 Ma ago), [18] (103 Ma ago) and [16] (94 Ma ago). Our divergence time estimates are the first that include *P*. *californicum*, indicating a late Eocene origin of this species (33.7 Ma ago). However, the age estimate confidence interval (25.1–42.6 Ma) largely overlaps with the age of Baltic amber of 35–50 Ma [27–28]. As a result, we feel confident to hypothesize that the *P*. *californicum* lineage may have already existed at the time of the Baltic amber forest. We cannot rule out that the Grolle & Meister fossil in fact belongs to this lineage. It is also possible, however, that this fossil represents a stem lineage element of *Ptilidium* or an early crown group species with morphological similarities to *P*. *californicum*.

Although little is known about the evolutionary age of bryophyte species and their morphological changes through time, an Eocene origin has previously been reconstructed for the leafy liverwort *Bryopteris diffusa* (Sw.) Nees [57,58]. Separation of the liverwort *Haplomitrium gibbsiae* (Steph.) R.M. Schust. from the rest of the genus possibly occurred at some point during the Permian [15]. On the other hand, speciation and extinction events are not necessarily visible in the fossil record or in chronograms based on DNA sequence variation of extant species. Such speciation events imply younger species ages than seen in the chronograms, but can only be reconstructed if a dense fossil record is available [59] or if fossil DNA is still accessible [60]. With regard to *Bryopteris diffusa*, the Miocene amber fossil *Bryopteris bispinosa* Grolle is of special interest [61]. Both taxa share a toothed lobule, and thus may belong to the same lineage. However, the fossil can be interpreted as a Miocene *B*. *diffusa* only if fundamental changes in the morphology of this species have occurred during its evolutionary history. Accepting the fossil as an extinct sister lineage of *B*. *diffusa* might imply a younger age of this species than seen in chronograms considering only the extant diversity.

In the absence of additional *Ptilidium* fossils we are unable to dismiss an Eocene origin of *P*. *californicum*, yet we acknowledge the uncertainties connected with this assumption. All other published Baltic amber fossils of liverworts have been assigned to extinct species. Conspecificity of the extinct *Nipponolejeunea europaea* Grolle with the extant *N*. *subalpina* (Horik.) S. Hatt., as suggested by [39], has subsequently been rejected based on divergence time estimates indicating a late Miocene or Pliocene origin of *N*. *subalpina* [13]. As a consequence, the missing *Ptilidium* fossil of Grolle & Meister [39] represents the only Baltic amber inclusion of a liverwort with a possible affiliation to an extant species.

### Tetralophozia groehnii

*Ptilidium* is an isolated genus that has been interpreted as an early diverging lineage of the liverwort order Porellales [62] or a member of the Ptilidiales [63]. Porellales/Ptilidiales are characterized by a complement of several structural features, including incubous foliation, exclusively lateral branches, and fasciculate rhizoids. Specimen GPIH 4575 [43] cannot be attributed to *Ptilidium*; rather, the succubous leaves and diffusely distributed rhizoids are suggestive of affinities to Jungermanniales, a generalistic main lineage of liverworts that originated in the Paleozoic [15,16]. Ten fossil representatives of Jungermanniales have been recorded for Baltic amber to date [39,64–66]; however, GPIH 4575 is not related to any one of these fossils. The rigid stem, brown colour, succubously transverse quadrifid leaves (Fig 2A and 2B), canaliculate leaf lobes (Fig 2D) and bifid underleaves (Fig 2E) correspond to the extant genus *Tetralophozia* of the Scapaniaceae sensu [62] and [67], or Anastrophyllaceae sensu [68]. Divergence time estimates provide some evidence in support of an Eocene origin of *Tetralophozia* [16,67], and thus do not contradict the morphology-based classification.

*Tetralophozia* includes four extant species that thrive in temperate and tropical Asia, Africa, and the Holarctics [50,69–71]. Molecular phylogenies [67] suggest that *Tetralophozia* and the morphologically closely related genus *Plicanthus* R.M. Schust. may be synonymous; however, current sampling does not suffice to resolve the actual relationship between the two taxa. *Plicanthus* is the younger taxon, and is separated from *Tetralophozia* by 3- rather than 4-lobed leaves and freely ciliate leaf margins. *Plicanthus* currently includes four or five species with a Paleotropic distribution [72].

*Tetralophozia groehnii* (Figs 2 and 3) represents the first fossil evidence of the genus *Tetralophozia*. The fossil differs from the extant species by the relatively regular dentition of the leaf lobes and teeth with a 1–4(5) celled uniseriate tip.

## Conclusions

The two Baltic amber inclusions initially assigned to the boreal species *Ptilidium pulcherrimum* in fact do not represent this species, and thus do not challenge the phytogeographical interpretation of the subtropical or warm-temperate Baltic amber flora. The specimen published by Frahm & Gröhn [43] is a jungermannialean liverwort, representing the first fossil record of *Tetralophozia*. The second inclusion, described in Grolle & Meister [39], may represent an extinct crown group element of *Ptilidium* or a representative of its stem lineage. Alternatively, this fossil might belong to the extant *P*. *californicum*. If the latter is correct, then this fossil represents the only Baltic amber fossil of a liverwort with a possible direct relationship to an extant species.

In spite of the dedicated work by Grolle [39], the inventory of liverworts in Baltic amber remains incomplete. Only three species have been added to this inventory since the publication of the monograph by Grolle & Meister [39], i.e. *Cephalozia veltenii* T. Katag. [66], *Notoscyphus balticus* Heinrichs, A.R. Schmidt, Schäf.-Verw., Gröhn et M.A.M. Renner [65], and *Tetralophozia groehnii* (this paper). Liverworts preserved in amber likely were epiphytes on the resin-exudating gymnosperms, or lived in the immediate vicinity and became embedded in resin trickling or dripping from trunks and branches. Epiphytes that prefer angiosperm bark, as well as terrestrial species growing in some distance to the resiniferous gymnosperms are rarely preserved in Baltic amber. Wind (e.g. during storms) and water (e.g., during floods) transport of liverwort fragments into resin flows may occur, but is exceedingly rare. Based on the fact that the diversity of epiphytic liverworts in present-day gymnosperm forests is usually lower than that in angiosperm-dominated forests [73–76], it is reasonable to conclude that only a minor proportion of the liverworts of the Baltic amber forest has been documented to date.

## Supporting Information

S1 File Agreement for use of the previously copyrighted image of the *Ptilidium* fossil for Fig 1A. (PDF)
