# Supplementary material for: Molecular and Morphological Evidence Challenges the Records of the Extant Liverwort Ptilidium pulcherrimum in Eocene Baltic Amber
Source: PLoS One. 2015 Nov 4;10(11):e0140977. doi: 10.1371/journal.pone.0140977 (PMC4633292; doi:10.1371/journal.pone.0140977)
Supplement: S1 File — (PDF) [file pone.0140977.s001.pdf]

WEISSDORN-VERLAG JENA
Dr. Gerald Hirsch
Wöllnitzer Str. 53
D-07749 Jena
Tel./Fax (+49)-(0)-3641-396584
www.weissdorn-verlag.de
eMail: weissdorn-verlag@web.de

Weiss dorn

Prof. Dr. Alexander Schmidt
Department of Geobiology
University of Göttingen
Goldschmidtstraße 3
37077 Göttingen
Germany

Jena, September 22nd, 2015

Dear Prof. Alexander Schmidt,

I hereby affirm that I, Dr. Gerald Hirsch, the original copyright holder for the previously copyrighted overview image of the fossil liverwort *Ptilidium pulcherrimum* published in the book entitled "The liverworts from Baltic and Bitterfeld amber" by R. Grolle and K. Meister (ISBN 3-936055-04-1), agree to re-publish that image in the work entitled "Molecular and morphological evidence challenges the records of the extant liverwort *Ptilidium pulcherrimum* in Eocene Baltic amber" by Heinrichs *et al.* (= Figure 1A) in the journal PLoS ONE under under PLoS' CC BY 4.0 license.

Sincerely Yours,

Dr. Gerald Hirsch
Weissdorn-Verlag Jena
Copyright holder
